# Online interventions and virtual day centres for young people who use drugs: potential for harm reduction?

**DOI:** 10.1186/s12954-023-00847-1

**Published:** 2023-10-27

**Authors:** Matej Sande, Bojan Dekleva, Špela Razpotnik, Darja Tadič, Mija Marija Klemenčič Rozman, Jana Rapuš Pavel

**Affiliations:** Faculty of Education, University of Ljubljana, Kardeljeva ploščad 16, 1000 Ljubljana, Slovenia

**Keywords:** Young people, Drugs, Harm reduction, Online interventions, Virtual day centre

## Abstract

**Background:**

The methodological part of the large-scale study on the psychosocial distress of young people in Slovenia focused on vulnerable young people who use drugs and explored the potential of online interventions in harm reduction programmes. We looked at the needs of young people who, at the time of the research, were attending a virtual Discord day centre hosted by the DrogArt NGO or were involved in the organisation's other programmes. We explored young people's knowledge of online interventions, their satisfaction with them and the opportunities they offer for harm reduction programmes.

**Methods:**

The study used a qualitative methodology with a combination of deductive and inductive coding, and relied on framework analysis, 18 young people who had used drugs or had stopped using participated in the study. The inclusion criterion was a maximum age of 25 years. In-depth interviews were conducted with the young people, which lasted on average between one and a half and two hours.

**Results:**

The study showed the potential of online interventions, specifically the virtual day centre, which provide a safe and relaxed space for young people in the sample to meet and talk, which is accessible and where they feel welcome. Online interventions have also enabled some of the sample to engage in the ‘offline’ support types offered within the organisation. The main advantages of online interventions are seen by young people as being more ‘geographically’ accessible and more available during the COVID-19 epidemic. Online support suits some people because they can leave sessions more quickly and it is more informal, while others prefer it because of specific problems or difficulties, such as social anxiety.

**Conclusions:**

The results show the relatively high potential of online interventions in harm reduction programmes, as well as more broadly for young people with various psychosocial difficulties and who, for example, do not use drugs. These types of support allow quick contact with a professional or peer and facilitate contact with a support programme. Young people are still poorly informed about the support programmes available in Slovenia and would like more information. Thus, in addition to developing and upgrading the network of programmes, we need to focus on providing information to young people through channels that are close to them and can reach them.

## Background

Online interventions or online support for people who use drugs (PWUD) is not new in the field of harm reduction and has been around for a long time in various forms. Harm reduction organisations carry out web outreach work and offer information and counselling to people who use drugs through online platforms [1]. Such web-based interventions are also referred to as ‘online outreach’ or ‘netreach’ [2]. Online forums, which are part of netreach work, have the potential to bridge the gap between professional and peer-based harm reduction initiatives [3]. In addition to forums, online drug interventions can also include various apps, self-assessment tests, etc. (ibid.), use reduction programmes and online self-help programmes or websites with provided contact channels [4].

Originally, online interventions under PWUD seemed more tailored to the subset of partygoers who attend electronic dance events [5], as this group has a better understanding of technology, but due to the increasing availability of technology and networking, they are now likely to be accessible to a wider range of people who use drugs. During the first wave of the COVID-19 pandemic in Slovenia, regular use of the Internet in the general population increased in all age groups, except for 16- to 24-year-olds, among whom it remained unchanged at 99%. In the first quarter of 2020, 58% of people in all age groups searched the Internet for health-related information, an increase from 48% in 2019 before the pandemic [6]. Despite the data on the relatively high prevalence of regular Internet use during the pandemic, HBSC research indicated that up to 7.3% of high school students had inadequate access (never, rarely, occasionally) to electronic devices and online tools for schoolwork and communication with friends during their senior year. More than a fifth of Slovenian fourth graders talk more easily about their feelings and worries on the Internet [7]. The HSBC study points to the potential for digital exclusion, as access to the Internet is not given or online interventions are not easily accessible or relevant to everyone.

Even before the pandemic, the role of the Internet in buying and selling drugs on various cryptomarkets had been increasing over the past decade [8], coinciding with the popularisation of NPS^1^ use in the post-2010 period. The internet was an important medium for harm reduction information regarding drugs and related issues for electronic dance event attendees. In research conducted in 2018 and 2014, respondents indicated that they would prefer to receive additional information (about STIs or NPS) via the internet or mobile apps [9, 10]. Our 2001 and 2005 research on the same target group also pointed to the importance of harm reduction information on the Internet, as well as online counselling and peer support in online forums [11].

Young people^2^ are a specific group that is highly skilled in the use of various apps and platforms. According to a survey [12], young people see the main benefits of online support in anonymity and privacy, ease and immediacy of access, and connecting with others who share the same experiences. Seeking online support can be a step on the path to seeking further support and can provide a sense of increased control over the process [13].

In Slovenia, some forms of online support started immediately after 1999 [14], when the first attempts at online counselling were made within the DrogArt Association,^3^ using email, chat software and an online forum, which has been running on its own web server. Among organisations where young people seek information about mental health online in Slovenia, DrogArt was ranked fourth in 2020 as the first ‘niche’ information provider specialising in drugs, sexuality and nightlife within the broad field of psychosocial distress [15].

The COVID-19 pandemic has also affected access to services for PWUD due to the constraints of public life, prompting programmes to use mobile or online platforms to mitigate the loss of face-to-face contact [16]. In Slovenia, during the pandemic, we observed significant limitations in access to health and social care services among young people who use drugs and changes in the functioning of some organisations offering various types of support to young people towards the implementation of online interventions [17]. During the pandemic, a day centre, ‘Dnevna soba’ (the Common Room), was launched at the DrogArt Association for young people who use drugs aged between 15 and 29. The day centre acts as a safe space where young people can socialise and participate in various activities, and various types of psychosocial support are available. Due to pandemic-related restrictions on movement and services, a virtual day centre (VDC) [18] was designed as an upgrade in 2020, which operates on the Discord^4^ social platform. Discord was chosen because it is a popular online platform for young people in Slovenia, offering opportunities for interaction, moderation and activities, and it is free to use. There was also positive feedback from other (non-drug-related) organisations on the use of this platform for VDC. The VDC operates on weekdays and features various activities such as a book club, a debate club, creative and fun activities, and socialising. Throughout its operation, the VDC also provides a chat room or group chat for young people, which also allows them to contact a professional and provides psychosocial support. In Slovenia, the digital youth centre DigiMC (on Discord platform), set up by the public institution Young Dragons, has also been launched during the epidemic.

In this article, we focus on vulnerable young people (with a history of drug use, mental health problems or psychosocial distress) who were in contact with various harm reduction activities of the DrogArt NGO at the time of the research and their perspectives on online interventions. We were interested in what kind of online interventions and online support programmes they are familiar with and what are the characteristics of such support, specifically VDCs. In addition, we wanted to assess the potential of online interventions and discover the possibilities that such forms of work can offer to harm reduction programmes.

## Methods

The methodological framework described in this article was part of a larger research project support networks of youth in psychosocial distress [19], which was based on a preliminary study [20, 21]. The project focused on vulnerable young people who use drugs, their support-seeking pathways or trajectories, and their knowledge of online interventions or online support programmes. The main objective of the methodological part of the study, which analysed a group of vulnerable young people with a history of drug use, was to examine the characteristics of the help-seeking process and to find out what works for them in seeking and receiving help and what provides them with adequate support. The specific aim was also to explore the potential of online forms of help and to find an answer to the question of how these programmes are tailored to young people. Only the results related to this aim are presented here.

The main research questions of this methodological part related to online interventions were: Are they aware of online interventions or assistance programmes that provide online support, and how relevant is this support to them. Other research questions related to the ‘offline’ support channels and the main difficulties in seeking help, but they are not presented in this paper. In this separate methodological part, we used a combination of deductive and inductive modes of analysis and coding, and the results presented in the article refer only to online forms of support. Given that we were already somewhat familiar with the field from our previous study on youth psychosocial distress and access to help, we relied on a framework analysis [22, 23], where we developed a typology of responses based on the research questions in advance, and later added more in-depth analysis using an inductive approach for each category.

### Participants

The part of the survey on vulnerable young people who use or have used drugs involved 18 young people who were involved in various ways in DrogArt's programmes or who were in contact with the organisation's outreach workers at the time. Initially, the inclusion criteria were age below 25 years and socialising in open public spaces in Slovenia. Due to the movement restrictions related to the COVID-19 epidemic, which affected the conduct of the research, this condition was extended to include participation in the various programmes and activities for young people who use drugs that were being implemented at the DrogArt Association during the research period. The age of users of some DrogArt programmes is up to 29 years or higher. The inclusion criterion of age under 25 was established by the research team of the broader research project because we wanted to include young people as defined above.

### Sampling

Data collection took place from November 2020 to August 2021. Just over half of the interviews were conducted in person, while the rest were conducted by videoconference (Zoom and Discord) due to epidemic-related measures and the sampling methods employed. Young people were invited to participate in outreach work in an open public space in Ljubljana, in the online day centre, in the physical day centre or in the counselling service of the DrogArt Association. Most of them were involved through the Discord online day centre (7) and the counselling service (6), while the rest were involved in day centre and outreach work (3) or worked as volunteer or field worker in the organisation^5^ (2) at the time of the interview. In-depth interviews lasted on average between one and a half and two hours, with the longest interview exceptionally lasting seven hours. Two interviews were conducted in two parts because time ran out in the first session. Interviews were divided among three DrogArt staff members, i.e. each participant was interviewed by one of the three members, who were part of the shortlisting team for the survey on young people who use drugs. The interviews were recorded with the participants' consent and later transcribed into stand-alone documents for further processing.

The study employed a semi-structured interview, which was developed by a small research team based on the initial research questions. It consisted of four thematic strands, which did not follow the same sequence in all interviews. Each strand contained various questions, which could be added to by the interviewers, who did not necessarily follow a set questionnaire. Special care was also taken to ‘translate’ the questions into the language of the young people. The interviewer, together with the participant or independently after the interview, also wrote down basic information about the participant in a separate questionnaire, which, in addition to basic demographic questions, also included questions about other particularly prominent and potentially threatening factors in the family, mentions of various organisations offering support and difficulties in accessing support. The portion of the interview that aligned with the research question consisted of questions related to online help, such as, Do you know of any organisations that offer online help? Have you used them before? How and through what channels would this help reach you? What would be your reasons for choosing or not choosing this type of help?

In accordance with the Code of Ethics for Researchers at the University of Ljubljana, all interviewees were provided with an informed consent to participate in the study, which included basic information about the purposes of the study, participation in the study, anonymity and publication of record, and risks. Participants were compensated for their time with €10, which we felt was a reasonable amount and not so high as to ‘force’ participants who might not otherwise have been motivated to take part in the study [24]. One of the ethical dilemmas that we raised when we were preparing the research design was that the interviewers may have had a consultative relationship with the interviewees, which could have influenced the results. They may have had prior information about the participants which they could have highlighted with questions during the interview. This dilemma was avoided by not interviewing users with whom the interviewers were in a counselling or therapeutic process or were providing psychosocial support.

### Data analysis

Starting from the research questions of the entire methodological strand, we used a deductive approach to create a basic framework of six categories (one of them was ‘Online types of support’, which is presented in this article) that we searched for in the text.^6^ After a more in-depth reading of the texts, in all interviews, we assigned the codes from the table to each part of the text, thus grouping them into the described content strands and adding subordinate codes (e.g. ‘potential of online interventions’ and ‘knowledge of online forms of support for young people’). Within each strand or sub-strand, we then inductively imputed more detailed codes by re-reading the texts, which we did not set in advance, but meaningfully imputed as we read the text (e.g. ‘suggestions on how to facilitate young people's access to online support’ and ‘limitations of support via social networks’ for the supportive relationship mentioned above). The codes have been given more prominence, and when they appear in the results section, they are marked in **bold** (for example: Facebook and Instagram through advertising).

## Results

### Sample characteristics

The sample consisted of 18 young people aged 16–25 at the time of the interviews. Most (5) of the participants were aged 20 years, and the average age of the sample was 20 and a half. There were eight males and nine females in the sample, and one person in the sample identified as non-binary.

The basic characteristics of the sample, such as socio-economic status, residence at the time of the study and the occurrence of various risk factors in the family and psychosocial distress of the participants, were checked by means of an accompanying questionnaire, which was written down by the interviewer at the end of the interview or together with the interviewee. Regarding sexual orientation, one-third of the sample identified as heterosexual, just under one-third identified as other and just under one quarter identified as bisexual. The remainder listed queer, pansexual, asexual or wrote that they did not identify or did not know yet under ‘other’. Two people identified as lesbian and one as non-binary.

At the time of the distress, the vast majority (15) of the young people in the sample were living with their primary family, with one person living independently, in a boarding school, and with their mother and stepfather. Three quarters of the participants came from a ‘non-normative’ family, and most of the sample had divorced parents or a single parent. Half of the interviewees in the sample lived in families with an average socio-economic status, while one-third rated the status as below average (poverty or material hardship). Three interviewees rated their family's socio-economic status as above average.

The sample had a range of mental health problems and distress at or before the time of the interview. Most (10) reported depression, self-harm (6), anxiety (6), suicidality or attempted suicide (4), panic attacks (4) and anxiety (3). In addition, other distresses, mental health problems, disorders or circumstances were reported, such as: self-punishment, eating disorders, personality disorders, sexual and physical violence, self-disclosure as a non-binary person, alcoholism in the family, statement of special educational needs, obsessive–compulsive disorder, acute schizophrenia, neglect, experience of homelessness, running away from home and hyperactivity. There was no one in the sample with no expressed distress or who expressed only a single distress or problem. All study participants reported one or more forms of mental health problems or psychosocial distress at the time of the study. In the past, the majority reported three or more forms of problems/stress (e.g. difficulty with disclosure as a non-binary person, excessive substance use, depression in one case and diagnosis of special needs, neglect, self-harm, homelessness in the other case).

Analysis of the accompanying separate anamnestic questionnaires shows that the vast majority of young people in the sample sought help from various organisations. Almost three quarters of participants have already sought help or been referred to a specialist—a psychiatrist (outpatient treatment)—or to a non-governmental organisation. A large proportion (11 of 18) had received treatment at school or at the school counselling centre or social work centre (10). Half of the participants in the sample were treated in a psychiatric hospital. One-third were treated by their primary care physician for problems. A relatively large number (7 of 18) sought help on their own from a private psychotherapist and two from a licenced psychotherapist. Slightly less than a quarter of the young people were placed in a reformatory or residential group. Three individuals in the sample had previously participated in a special programme (other than DrogArt) to support people who use drugs.

### Characteristics of drug use

According to the purpose of the research and the sampling method, our sample included young people who use or have used drugs and who sought contact with the organisation, or were involved through outreach work in an open public space in Ljubljana.

The frequency of use and the issues associated with drug use vary widely among the people in the sample. Just under a quarter use drugs relatively **frequently or regularly:** *"Well, it comes and goes, but most of the time I smoke weed, even if it's bad for me, even if it makes me paranoid. Quite a lot, definitely at least twice a week, sometimes every day."* (INT 1).

A minority of respondents report **occasional use, e.g.** a few times a month or less *… ‘Now, during the holidays, I use weed … maybe like 1* ×*, 2* ×*, 3* × *a month on average. I don't have as much time now that I'm working. But it also depends on how much time my friends have (laughs). I don't smoke alone, in any case’. (INT 10) …* up to a few times a year or occasionally.

A large part of the respondents had experience with various drugs in the past, such as stimulants, psychedelics or depressants, but had **reduced their use:** *"I used to use weed quite a lot in the past, … Then I got into cocaine as well. But now I'm off cocaine, and I only use weed when I really can't sleep." (INT 8).*

Some describe **risky drug use**, e.g. **a combination of licit and illicit drugs:** *"Well, I'm on antidepressants at the moment … I'm on minimal doses of Xanax, Quentiax, Lyrica and Propanolol. I'm just taking those to help me sleep, apart from Propanolol. These are minimal doses, like 0.5 Xanax, 25 mg of Quentiax, and about 150 of Lyrica, which I guess isn't really a minimal dose, but it's also not that big of a dose either. Some Tramal for when I have back problems … Otherwise, yeah, as far as doses go, I smoke two or three joints per day. Last week, when I had extreme back problems, I did a little bit of ketamine three times, since it is an anaesthetic, after all (laughs). Um … Other than that, I don't know, every roughly two months, I treat myself to a little bit of some substance or other, but other than that, not really." (INT 7),* or **injecting heroin.**

Almost one-third (5) of the sample were young people who had either **stopped using illicit drugs and alcohol (4)** or **used** them **relatively sparingly (1).** One of the participants who stopped using drugs gave the reason that he/she was being drug tested as part of a special support programme and only consumed alcohol (usually binge drinking), while the other gave panic attacks as the reason for quitting. As mentioned earlier, three participants had previously been **involved in a specialised support programme** for people who use drugs One of them stopped taking drugs for the above reason (drug testing).

### Knowledge of online forms of support for young people

In this section, we present the results of the qualitative survey on online support. We also addressed this issue in the quantitative survey (as part of the large-scale study mentioned in the Methods section), and some results are presented in the discussion. The majority (three quarters) of the young people in the sample were familiar with DrogArt's forms of online support, which is understandable as we also sampled its programmes. Four participants were not aware of any online support options: *"No, I don't know. I'm not sure about [name of org.] either, I think they do have it, but I don't know."* (INT 13), and of these, one participant involved in the counselling service was not skilled in the use of the internet since he did not have access to it during his stay in various institutions: *"I mean, as far as being skilled in the internet goes … the thing is, I wasn't even allowed to have a phone in institutions, much less a computer. Some places would let you have a phone but not a computer. You could have a phone for only a limited time, at certain times of the day. In mental institutions, you were usually allowed to have a phone for … like one hour in the evening at home. I don't think we were allowed to have computers, but I did see one person who had a laptop. And the hospital usually has a laptop … well, not a laptop, but a PC that people can use after dinner or something." (INT 17)* From the quotes, we can see that there is still a gap in terms of access to the Internet among the young people in our sample, e.g. limited access at the institution or lack of information about online services.

In addition to DrogArt, four young people knew of other organisations offering online support. One of them only knew of a foreign service (online therapy), which later turned out to be fake: *"Yeah, I know an American one that was promoted by YouTubers. Some sort of online therapy, but it's for a fee, that's the problem. The ad was that you definitely get a specialist with every text message and every call. But then it turned out to be fake all along, and very disorganised. Nobody really looked into the service that was being advertised."* (INT 1).

Two were familiar with **Young Dragons (Digi MC)** and one interviewee mentioned the **psychosocial counselling service at the college** by video call and the option to work with her **therapist** online. Thus, knowledge of the various forms of help was relatively low in our sample. As mentioned earlier, Digi MC was established as VDC shortly before DrogArt VDC, and the online psychosocial counselling service was offered at the Faculty of Education, University of Ljubljana, and was online only during the COVID-19 pandemic.

One young person also mentioned ‘self-help courses’ on YouTube: *"There's a lot of that on YouTube already, people are helping themselves these days. If you can't get help, you can watch a million videos of therapists analysing things on YouTube or people with autism talking about their experiences, and you can relate that to your own experiences. If nothing else, you can find help on your own …"* (INT 12). YouTube was specifically mentioned in five of the interviews as a medium for skill sharing, “fake counselling”, or as a way to advertise online forms of support. Given that the young people in our sample use videos to seek information or help on a variety of topics related to psychosocial problems, this could represent potential for online support in terms of outreach and first contact with the target audience. The participant also mentions forums she used in her teenage years as a form of ‘online self-help’.*»Actually, I used online help quite a lot in my early teens. I was mainly motivated by anonymity. To be able to write whatever I wanted and even pretend to be another person, if that somehow satisfied my needs at some point, and no one could reveal this secret identity of mine … I posted on forums when I was young."* (INT 2). The forums may be losing importance in favour of other forms of online communication, but they were important ‘spaces’ for online help and information on various topics, provided moderation and mutual support, and some of them are still active in Slovenia.

### Where can we reach young people online and what are some suggestions for online forms of support?

We wanted to know how to reach them most easily online. The vast majority of them mentioned that they could be reached on **Facebook and Instagram through advertising:** *"… And maybe a little more money for promotions, so that people actually see their posts, that there is help here, you can help yourself here. The problem is that these organisations simply don't have the money to be able to afford it …'"* (INT 6). Three of them also suggest **advertisements during the video content on YouTube**, advertising online support or, as in the following example, offering specific video content about coping with hardship: ‘ *… maybe even on YouTube, as I said before. And it doesn't need to be just a YouTube video. When you upload something, it can either direct people somewhere or actually provide support in the video itself. Something like ‘how to deal with a panic attack’, whatever, anything like that’.* (INT 12). Two also mention the **integration of various online forms of support:** *"For young people, I think social networks, definitely. Facebook, Instagram, I'm sure they'll be noticed there. As for [name of org.], I learned about it through [VDC]. So basically, everything is connected."* (INT 8).

Recognition of the organisation can also help online advertising, according to the interviewee: *"Since you do have recognition, I think people would notice …"* (INT 16), but they would be put off by the participation of online influencers when advertising support on social networks. *"When the face of the ad is someone who is obviously doing it purely for self-promotion or something. So it's clear that something else is at play here, they are not just offering support to people."* (INT 14).

One of the interviewees also mentions **the limitations of support through social networks**, which do not allow for a more personal approach, as, for example, with Instagram, the support is not linked to the names of the people providing the help, but to the profile of the organisation: *"But Instagram is, should we say, quite popular. But then there's the fact that you can't build a community on Instagram. You can give information on Instagram, you can promote events and things like that, but you can't have conversations. You can have someone write to you from an account, but I think that can even make it more difficult for some people. When the account is just Team [org. name], it's a lot more … you just don't know who you're talking to. You're just talking to an entity and that makes it weird. Discord, I think, is very useful for creating communities like that. Facebook is kind of dying out. Nobody uses it that much any more. Uh … and then there's also TikTok …"* (INT 9) Based on this quote, the discrepancy between social networks and Discord in terms of creating communities is clear. It also refers to the ‘impersonal communication’ through the organisation's social profiles. However, at DrogArt, online interventions via direct messaging (DM) on Instagram were used with considerable success and with increased interest during the COVID-19 pandemics. The response to DM was general, without specific introductions with personal names, but if the problem or need was significant, the online counsellor always introduced and guided the user in further steps in the process of seeking help. One participant feels disadvantaged because she does not use social networks. She does not intend to use them in the future because, according to her, she has abused them in the past. When planning interventions (or advertising), we need to take into account those young people who do not use or do not want to use social networks.

**Suggestions on how to make it easier for young people to access online support** include a **website or portal with all the information collected.** *The page should be properly designed and contain up-to-date, clear and easily accessible information on how and where to find help: ‘So, I think that it would be very nice if you could just google “How to find help in Slovenia” and receive clear information. Clarity is the goal. There is always a twist. I have never seen help clearly offered in Slovenian. Something like, “This is for everyone with mental struggles, if you call this number, if you text this number, you will get answers there and then”, and then get an actual follow-up. And something with a relatively recent date, not from, like, 2003. Because that's what I found, the first thing I found was, like, “Information”, and then at the end, the date of publishing was 2011. I mean, really? How am I supposed to know now if this still exists? What is the latest thing? There should be one page in Slovenian, which you could easily google … One page with all the information, that would be very nice. And all the things that exist in Slovenia, the communities, which ones are free, which ones are for a fee, and have those listed separately. It should be nice and clear, not all black and white but nicely designed. Graphic design is very important, people don't realise it, but it is. And the page has to work, of course. That's often a problem as well, when you click on something and it doesn't take you anywhere, or there's an “error”. And it's important that something is happening, that the site is alive, that it's not just whatever, that it's not 2011, you know.“* (INT 1) From the quote, it is clear that online support should be up-to-date, clear, easily accessible and provided in Slovenian. The web interface offering support or phone number should also provide feedback and follow-up. The participant also suggests a site that would offer various information on the way to adulthood, e.g. on schooling, loans, income tax: *"If there was one portal for young people where you could literally find everything-how to apply for college, how to apply for high school, how to calculate your loan interest, how to calculate your income tax, where to get assistance, … all of this on a single portal that is always accessible, open and has everything in one place-that would be optimal”. Because it is very difficult to use the Slovenian internet, if someone doesn't tell you in advance where to go, what to serach for. If I search for ‘online support’, I have a feeling that I won't find anything useful.*’ (INT 12) In addition to the web portal, they also suggested information on mental health support at faculties and traditional advertising on school bulletin boards. From their suggestions, we can summarise that we need to provide more than basic information about psychosocial distress and promote the online support offered on channels that are relevant to young people. We should also focus on the design and relevance of the information provided, and on responding quickly to their questions or need for support. In addition to suggestions for online support, they also suggest raising public awareness of psychosocial problems and reducing the stigma associated with them. They also suggest reducing the stigma of ‘recreational drug use’ or ‘past drug use’ among professionals who assist young people seeking help for psychosocial problems.

### Benefits of online types of support

The interviews highlighted various advantages of online types of support according to the participants. As later quotes show, young people stress the importance of ‘modernising’ organisations in the field of online support. They are growing up in a time when information is readily available, and they are ‘connected’ to the web through various devices for a large part of their time.

The young people in the sample see the usefulness of online forms of support, on the one hand, in the wider ‘geographical’ **accessibility of the support:** *"People who don't have access to your day centre, who live outside Ljubljana or in another country—I think online counselling would be very useful for them. I mean, you use Skype, right? Yeah, that would be really nice for people who don't have access to a live day centre. Well, actually, I did have a conversation with Simona [a counsellor] once during the quarantine and we couldn't meet in person, so we met over Discord." (INT 3).* Others say that such support was **available even during the COVID-19 epidemic**. *"I find it very useful in case you really can't go anywhere. Like with the current lock-down [quarantine]. I find it very useful, no matter if remote or in person, because you can open up … But I also like the online way, which you can use in case you're actually stuck inside, or you don't want to go somewhere, or you can't go somewhere. In those cases it's great."* (INT 8) And: *"Yeah, now during quarantine, if there's no access, if there's no exits, if you're not allowed to go out, the [org. name] Discord channel is great. But I still prefer to visit in person when that is an option."* (INT 3). At this and other points, some interviewers mention the preference for or importance of personal contact. This form of support can be attractive to young people because it is simpler (more comfortable) and there is the possibility of **leaving or withdrawing quickly:** *"I always prefer online, since I find it easier than in person … It's easier for me when I'm not there. If something is wrong, I can turn it off in a second. But if you're there in person and something bothers you, you can't say anything, you still have to be there. Online, I can easily back out. And I'm at home, comfortable in my chair, warm and cosy, which is nice. When you get somewhere it' can be a bit awkward and you get a bit anxious and all that."* (INT 4) One of the interviewers described the advantage of more **'informal'** support in VDC and a relaxed ‘atmosphere’ that young people can more easily identify with: *"Yes, many. I see a lot of links, these things that are there, they're very useful. And also the stuff that happens on the voice call can be useful, although I don't really like the current ones, anime and books. Maybe books* [7]*, yeah, but I don't dare to join in anyway. But it's relaxing, I like that a lot. It's noting formal, I like that too, so it's easier to relax. And young people are like that, you know, they are growing up, they're young, they don't like all that formal stuff. It scares everybody, at least me personally. I like that there's a relaxed vibe, people are chatting normally, everything's clear, and you know where everything is. I like that a lot."* (INT 4) Three participants say that this format is more suitable for people who are **socially anxious:** *"And it's also nice for socially anxious people, so that they can get a chance … Since they couldn't otherwise, because they are self-conscious. Or they are … I don't know, too nervous or something to actually approach the organisation physically. I think that's a big factor here. Because not only can you offer better support, but you can help more people."* (INT 9), and: *"I would have chosen to do it myself simply because I'm much more comfortable being at home and online. I'm still overcoming my social anxiety, so being on the computer by myself, even if there are a lot of people [online], it's a lot easier and it's a lot more comfortable. Well, I prefer it, in any case, to driving an hour to get support. So, it's very accessible to me, if it's online. Especially during the epidemic, there's no other option."* (INT 6) All participants state that this way is easier and more appropriate and that they are less likely to abandon the support programme: *"Yeah, but for a start, I think it's good if you're someone who maybe wants to try therapy, a little extra help … especially if you have anxiety, things like that, maybe it's easier for you to be online for the first couple of sessions … Just so you can see what it's like without having to go through this physical process that might prevent a lot of people from getting help … many people are like, ‘Ok now I'm going to go to therapy’, but then they have to worry about catching the bus, doing this and that, going here and there, and they just get overwhelmed by emotions and say ‘screw it’. But it's much easier online …"* (INT 12).

One respondent explains VDC's **usefulness** as an online approach that is close and accessible to young people::*"I think it's a very good approach, because young people are now growing up in the online age. So it's rare to find someone who remembers having a device that wasn't connected to the internet. And it seems to me that this culture is just so much more normal now that organisations just need to modernise in this regard. Which they are doing for the most part."* (INT 9) As mentioned earlier, the Discord server can also provide additional support and contact with peers when needed, as indicated by some participants: *“’”* “I feel like I can always get in touch if I need to. Right now, it was not so bad that I needed to do that. When I’m in a bad mood, it’s easier for me to just come and chat. They are all like me here. Young, with their own lives. That’s kind of cool.” (INT 4).

In terms of the limitations of online support, four participants mention that they **prefer face-to-face support to online support** due to the lack of contact. *"… it still helped me a lot, it was great, but if I have to choose, I prefer face-to-face. This is because I can be much more vulnerable face-to-face. When online, I tend to be more quiet, I feel like I'm not really there, and therefore I'm not in touch with my feelings … but that is really specific to me.«* (INT 12).

### The potential of online interventions

We were able to recruit some of the interviewees who took part in the survey on Discord, where the VDC Common Room has been hosted since the beginning of the COVID-19 epidemic, and where volunteers and staff of the DrogArt Association have been involved in the management and implementation of various activities (such as the ‘kafana’, debate club, book club, drawing, etc.). The interviews highlighted the importance of online interventions, which according to the interviewees are **accessible** and can represent a slightly different and perhaps easier way of accessing support. For example, one participant explains: *"There is always the possibility to talk to someone from [name of org.], I like that a lot, there is always someone available on voice chat. It's great for people in need, or if they're bored, or if they're lonely. But the problem is, if I had found it three years ago, before I started therapy, I would never have joined the voice chat. Because I had such social anxiety that I could just never do it." (INT 1).* Another participant also describes the accessibility or the feeling of accessibility of support: *"But I think it's really good to know that if I ever want or need support, I can go to this group." (INT 6).* The sense of accessibility is emphasised because the professional staff and others from the organisation who provide support on the server are not present all the time, but during the active hours. However, they can respond relatively quickly to the questions and needs of the people participating in the server.

Most of the young people in the VDC found this format very convenient. It is a place for them to meet, **talk and have a ‘safe space’** where they can also get the information they need. *"First of all, I like the fact that you can kind of hang out on the internet, and that you can get answers for everything, whatever you need, everybody is there, they know everything, so it's really a safe space, it's very chill." (INT 4).* Online support can also be experienced as **more relaxed and less formal**, where open and honest discussions on various topics (including drugs) are possible. *"Not being formal, I like that a lot, it makes it easier to relax."* and *"I feel so relaxed on this server, I can be myself, I don't have to hide, I can be honest, even if it's about drugs. Other people don't want to hear about that or they freak out. So it's a very relaxed atmosphere. It's nice to have a little place on the internet like that. (INT 6).* Interviewees also mentioned that they felt **accepted** in the online day centre: *"I feel accepted here because I see that there are other people with similar problems. And I feel like I fit in." (INT 8).* In addition to feeling accepted, they also feel a **sense of community**, which helped, for example, to overcome loneliness during the COVID-19 epidemic: *"I like it so much as a community … It was great particularly during the lock-down, to help fight off the loneliness. (INT 11).* and *"… the community is great. Everyone is so nice. You immediately feel accepted. I really like it here." (INT 10).*

Some also enjoy the **activities of the online day centre**. *"I saw it, I joined and I liked it, so I stayed and now I've started to follow it a bit more closely, I like to go to the workshops and it's really nice. It's like being active, you meet new people. I really like it." (INT 5).* Online interventions can be the first step towards further support. For example, one person reported that initial contact was made through Instagram (information about the VDC), then through the VDC, and later she came to an ‘offline’ counselling service provided by the same organisation. I think I saw the information about the Common Room on Instagram, and the counselling was through the Common room. I got some additional information, some more information from you, and I said, Damn, that sounds good. I might try that out" (INT 7). The interviews revealed the benefits of online forms of support through Instagram and the Discord day centre. In addition to accessibility and the aforementioned suitability to the young people from the sample, online forms of support can also represent a first contact with support or an organisation and later allow for the inclusion in further support and counselling.

## Discussion

The results of our research demonstrate the benefits of online forms of intervention and their potential for use in harm reduction programmes. The results are consistent with findings from other research on the utility of online platforms in harm reduction contexts in the way that they provide insights into drug use trends [3, 25] or have potential for web-based outreach work [1].

To our knowledge, our study is the first to examine VDCs for PWUD on the Discord platform and the benefits of such ways of working. The results of the study need to be understood with the limitations of qualitative research and the relatively small sample of young people who were involved in harm reduction programmes implemented at DrogArt at the time of the study. Despite these limitations, the results can provide us with important insights into the mindset of young people with drug use experience and their evaluation of online interventions at a time when the internet has become an indispensable part of our lives. The high visibility of the organisation's online interventions in our sample is understandable, as we sampled across the organisation's programmes. The results of the survey, which was part of the second methodological strand of our broader research on a larger sample of 1,143 young people [15], showed that DrogArt is ranked fourth among 17 providers of mental health content for young people in Slovenia, and first among specialised programmes offering specific information, the vast majority of which is drug-related. This shows the organisation's good online reach and complements the qualitative results.

Despite the above, our study found that young people are relatively poorly informed about the support programmes that offer help for mental distress and would like more information. Less than one-third of the young people in the sample were aware of other online forms of support or information besides DrogArt. They suggest non-judgemental awareness-raising about support and the importance of mental health, as well as the availability of programmes for a wider range of psychosocial distress than just drugs. In addition, they suggest more ‘publicity’ for programmes offering support to young people. This publicity should be tailored to their target group using appropriate communication channels, of which most participants propose online advertising. They also suggest raising awareness of mental distress among the general public and reducing the stigma attached to it. In their view, the best way to reach young people is through advertising on social networks (Especially Instagram, since Facebook is losing popularity among young people [26]). There was also a proposal for a Slovenian portal on mental distress and for information on mental distress in faculties and schools, possibly in the form of traditional advertising.

The main advantages of online interventions are seen by young people as being more ‘geographically’ accessible and more available during the COVID-19 epidemic. Online support suits some people because they can leave sessions more quickly and it is more informal, while others prefer it because of specific problems or difficulties, such as social anxiety. This relates to the findings of a study on web outreach work [1], which showed an increasing level of efficiency that comes from online provision of harm reduction services in terms of easier and faster accessibility. Online interventions are not for everyone, however, as some participants indicated that they prefer face-to-face contact and may be even more anxious online than in person. The study was also conducted during the time of the COVID-19 epidemic, when the shift to online education and other online services was quickly becoming the norm, and this may affect the results of our study.

The study showed the potential of VDCs to provide the young people in the sample with a safe and relaxed place to socialise and talk, which is accessible to them and where they feel accepted. The participants liked the activities of the day centre and the availability of professionals during the activities or their quick responses when they were not available on the platform. In addition to the quick accessibility and involvement in the programme, the VDC also allowed some in the sample to engage in ‘offline’ forms of support within the organisation. Such a platform thus offers young people who have not previously been in contact with the organisation to get involved in a way that is easier for them, and to enter other harm reduction services at a later stage. VDCs proved to be of great importance during the COVID-19 epidemic [27], as they provided access to assistance when it was no longer physically possible. Even after the epidemic, the DrogArt VDC allowed young people who were suited to this approach of ‘hanging out’, participating in activities and having access to information and psychosocial support, as well as one-to-one chats with professionals. The VDC is currently still operational, but on a smaller scale than during and after the epidemic, due to the increased face-to-face work of professionals in the organisation and operation of the day centre. The study did not evaluate the effectiveness of VDS, but rather its acceptability to young people. It would be good to explore their effectiveness in future work and compare them to traditional face-to-face day centres.

The results presented in our study are a summary of young people’s opinions or perceptions of online help. To put these more descriptive results in perspective, much more work and more in-depth analysis is needed. This includes interpreting what young people mean and how they connect the qualities of the service with those of the service design and online platforms they refer to.

One of the interesting findings of our broader research is also that the area of illicit drug use is in many ways not an independent area isolated from other areas of youth problems. The young people in our sample had many different mental health and psychosocial problems in addition to drug use. There may be some disconnect between how professionals or harm reduction agencies view these types of problems and how young people perceive them. Often they seek help for other problems in other agencies or programmes that have nothing to do with drugs, and the help offered may discourage them from continuing to participate. Or they look to harm reduction programmes (e.g. DrogArt) for solutions to their problems that are not primarily related to drug use, as these are more accessible to them and the waiting time for counselling is shorter. In designing online and offline support networks that provide help for a broader range of problems that trouble young people, we need to be aware of these issues and find appropriate responses tailored to their needs.

## Conclusions

The results of our research have shown the relatively high potential of online interventions that can be implemented in harm reduction programmes to increase reach and ease entry into services. Such ways of working are suitable for young people who are difficult to reach or who are less motivated to participate in support processes and prefer an informal approach. The VDC has proved to be a way of working that suits young people well because of the quick access, the relaxed space to socialise and the rapid availability of professionals available to offer support. The VDC has also shown to be an advantage as an entry point into the organisation where they can later seek face-to-face support and advice. Although young people are relatively poorly informed about the programmes offering support in psychosocial distress in Slovenia, they would like to see more information not only relating to drugs but to a wider range of psychosocial distresses. In developing and upgrading the network of support programmes, we must also pay attention to providing information to young people through channels that are close to them and can reach them.

## Data Availability

The data used for this study is not publicly available. For further information on the data and materials used in this study, please contact the corresponding author.

## Abbreviations

PWUD: People who use drugs
VDC: Virtual day centre
NPS: New psychoactive substances

## Footnotes

1 New psychoactive substances.

2 We refer to the term young people or youth as defined by the United Nations, ages 15–24.

3 DrogArt Association focuses on harm reduction related to drugs and alcohol. The main activities and services are harm reduction at electronic dance events, counselling and psychotherapy, drug checking, psychosocial support, day center for young PWUD and outreach work.

4 Discord is a social platform available as an app (for cell phones and web browsers) that enables real-time voice calls, video calls, and messaging. The platform provides the ability to build communities (also called servers) with "chat rooms." The platform is useful for creating VDC with different forms of communication (public or private chats), channels (rooms) and activities.

5 At DrogArt, young people with substance use experience can volunteer at selected activities in the organization to promote their inclusion in work processes and integration.

6 Other categories were, for example, help-seeking process, relevance of help provided, constraints on help- seeking.
